# Living with glaucoma: a qualitative study of functional implications and patients’ coping behaviours

**DOI:** 10.1186/s12886-015-0119-7

**Published:** 2015-10-06

**Authors:** Fiona C. Glen, David P. Crabb

**Affiliations:** Division of Optometry and Visual Science, School of Health Sciences, City University London, Northampton Square, London, EC1V 0HB UK

**Keywords:** Glaucoma, Visual functioning, Visual field, Interviews, Coping, Adaptation, Qualitative, Low vision

## Abstract

**Background:**

Sight loss from glaucoma can have a significant impact on functioning and performing everyday activities, but this varies between patients. The purpose of this study was to explore whether patients with glaucoma use different coping strategies in response to their vision loss.

**Methods:**

Audio-recorded semi-structured interviews were conducted with 16 patients (median age: 71 [interquartile range [IQR]: 68 to 77 years]; 50 % female) about their experiences of living with glaucoma. Patients had their glaucoma diagnosis for at least 5 years (range: 6 to 29 years) and had a range of disease severities (median best eye Mean Deviation was −9.1 dB [IQR: −12.9 to −4.1 dB]). A framework approach to analysis was taken whereby data was indexed using manual and computer-assisted methods, with codes applied to depict areas of functioning perceived to be impacted by glaucoma and coping behaviours used in response to these difficulties.

**Results:**

In order to maintain independence, some patients increased confidence by making practical changes such as adjusting lighting, using handrails and magnifying glasses, or actively changed aspects of their behaviour such as moving their head and eyes towards known areas of vision loss. Support from friends and family was often used, although some people worried about becoming a burden. Others imposed self-restrictions or gave up activities, thus compromising well-being and independence. Certain coping strategies were linked to time since diagnosis and location of vision loss. The type and quality of information received during clinical appointments, and the potential benefits of communication with other patients, emerged as other important themes.

**Conclusions:**

Results from this qualitative study suggest that the adoption of certain coping behaviours and techniques may help some glaucomatous patients to adapt to their condition. An awareness of coping and adaptive strategies, in addition to the usual clinical tests, may provide a better insight into the impact of disease and help inform future educational and management strategies for glaucoma.

## Background

Glaucoma can lead to gradual and permanent loss of vision in the visual field (VF). Once diagnosed, patients require lifetime treatment and monitoring of their vision to halt or reduce further disease progression. Glaucoma is often asymptomatic, particularly in earlier disease stages, meaning detection is difficult [1]. As the disease progresses, however, many patients experience visual symptoms which can have a significant impact on quality of life and ‘everyday’ functioning; this is supported by studies using patient-reported outcome measures and objective assessments of task performance [2–8]. Nevertheless, patients with similar clinical measures of disease severity and VF loss can report different outcomes. Performance-based studies of visual function in glaucoma have shown that some patients, even with quite advanced disease, perform well within the normal expected range, suggesting they may be responding differently to their vision loss, perhaps, for example, by changing eye movement behaviour [9–13]. Other studies support the view that some patients are more aware of the effects of their vision loss than others and this might precipitate the use adaptive strategies to deal with their sight loss [14, 15]. On the other hand, some patients may compromise their independence, instead choosing to limit or give up on activities, such as driving, as a result of glaucoma [16, 17].

The manner in which a patient responds to vision loss may be influenced by how well they adapt to their condition or develop coping behaviours [18]. Yet, there have been no studies, to our knowledge, that have directly considered the relationship between visual functioning and coping behaviours in patients with glaucoma. The current study therefore aimed to identify different strategies used by patients with glaucoma to cope with vision loss during everyday activities.

## Methods

Participants were recruited via advertisements placed in the International Glaucoma Association newsletter (www.glaucoma-association.com). Interested parties contacted the research team via email or telephone to receive the full information booklet for the study. A suitable time was then arranged for those still interested to travel to City University London to take part in the study.

Participants were required to have a diagnosis of Primary Open Angle Glaucoma (POAG) and to have been under glaucoma care in the United Kingdom for at least five years: these criteria helped to ensure that participants had sufficient experience of living with glaucoma and receiving treatment and follow-up care. They were also required to have measureable visual field (VF) loss in at least one eye (confirmed during a VF examination during the study visit). Volunteers were excluded if they had an ocular condition other than glaucoma (i.e. age related macular degeneration or diabetic retinopathy) or cortical visual impairment. Potential participants were also excluded if they had dementia or another isolated cognitive impairment. Participants were required to speak fluent English and provide their own informed written consent.

The study was approved by a Research and Ethics Committee (City University London School of Health Sciences) and adhered to the tenets of the Declaration of Helsinki. Data was anonymised and stored in a secure location. All participants gave their informed written consent prior to taking part.

### Pretesting

All patients underwent an examination of their vision by a qualified optometrist (including refraction, contrast sensitivity (CS), visual acuity (VA) and slit lamp examination on both eyes). VFs (Swedish Interactive Threshold Algorithms Standard 24-2) were measured monocularly, using a Humphrey Field Analyser (HFA) [Carl Zeiss Meditec, Dublin, CA]. Monocular VF measurements were then used to construct an integrated visual field (IVF), to be used as a surrogate representation of binocular visual function. Here, the best sensitivity at each VF location is used to construct a binocular VF [4, 19–21]. Participants also completed a measure of their cognitive ability (Mini-Mental Status Examination [MMSE]) to ensure their suitability for the study. Participants provided information about their general health (via EuroQol 5-Dimensions (EQ-5D) questionnaire and general information about other health conditions).

### Semi-structured interviews

Semi-structured individual interviews were conducted between November 2014 and February 2015. The interviews were audio-recorded (with the permission of the participant) and were based on a topic guide devised prior to the study that outlined broad question areas covered circumstances surrounding their diagnosis and how the disease has impacted them over time. Interviews followed a narrative approach. Patients were encouraged to describe aspects of their “glaucoma journey”, using a series of key questions relating to experiences at diagnosis through to the current day and how they approached any problems or difficulties relating to their vision. Questions were not leading; prompts were nevertheless used to encourage a participant to expand on what they were saying, or to clarify a question if it appeared that the participant had misunderstood the interviewer. Effort was made to ensure that participants were describing experiences relating to their corrected vision; that is ensuring, as far as possible, they were describing symptoms and circumstances that occurred when wearing their spectacles.

Participants were not given a formal copy of the topic guide, but they were told that there would be, “some discussion about your glaucoma and the circumstances around your diagnosis; we will also talk about how it impacts your life and how you deal with having glaucoma on a day to day basis”. Several participants voluntarily provided the researcher with written notes about their thoughts and memories relating to these topics prior to the study; this information was subsequently referred to within the interviews. It was emphasised prior to the interview that there were no right or wrong answers and the participant would be given the opportunity to expand or clarify any points at the end of the interview. The researcher took some field notes during the discussion.

All interviews were carried out face-to face, except for two carried out via telephone as the participants were unable to travel to London. The interviews were carried out by one of the authors (FCG), a post-doctoral researcher with experience of carrying out both qualitative and quantitative research involving patients with glaucoma. The researcher corresponded with participants during the recruitment process but had never met them in person before the study visit. Participants received financial reimbursement to compensate them for their travel costs and time. The study was aligned with the Consolidated Criteria for Reporting Qualitative Research (COREQ) [22].

### Analysis

A framework analysis was chosen for this study; this approach allows for a combined deductive and inductive approach to data analysis [23]. Audio recordings for the interviews, which lasted between 23 and 105 min, were transcribed verbatim by an independent transcription company. Transcripts were then combined and organised alongside additional notes taken during the study for each participant. The lead researcher (FCG) thoroughly read and re-read each transcript, and listened back to the audio-recorded interviews to ensure accuracy of the transcript and to re-familiarise herself with the whole data set. A subset of the transcripts was then read by another researcher (DPC), who was not present during the interviews or aware of the participants’ identities or visual histories. The team then manually worked through a selection of transcripts and, in light of the research question, highlighted segments of the text that corresponded to visual activities and aspects of functioning that were perceived to be affected by the interviewed patients. Different coping behaviours used by patients within these visual functioning areas were then identified. Certain behaviours were emerging consistently across categories, although there were initially some discrepancies with regard to terminology and assigning labels. For example, codes such as “moves eyes towards areas of poor vision” could also be coded at a higher level such as “actively changes behaviour”. It was ensured from this point that terminologies were consistent and that data was coded at the more specific level. Codes were also applied for additional themes emerging from the manuscript. For subsequent transcripts, Computer Assisted Qualitative Data Analysis Software (CAQDAS) [NVIVO V.10.2 (QSR International, Cambridge, Massachusetts, USA)] was used to apply and store the framework electronically and to identify any additional (unexpected) themes from the transcripts.

### Participants

**Fig. 1:**
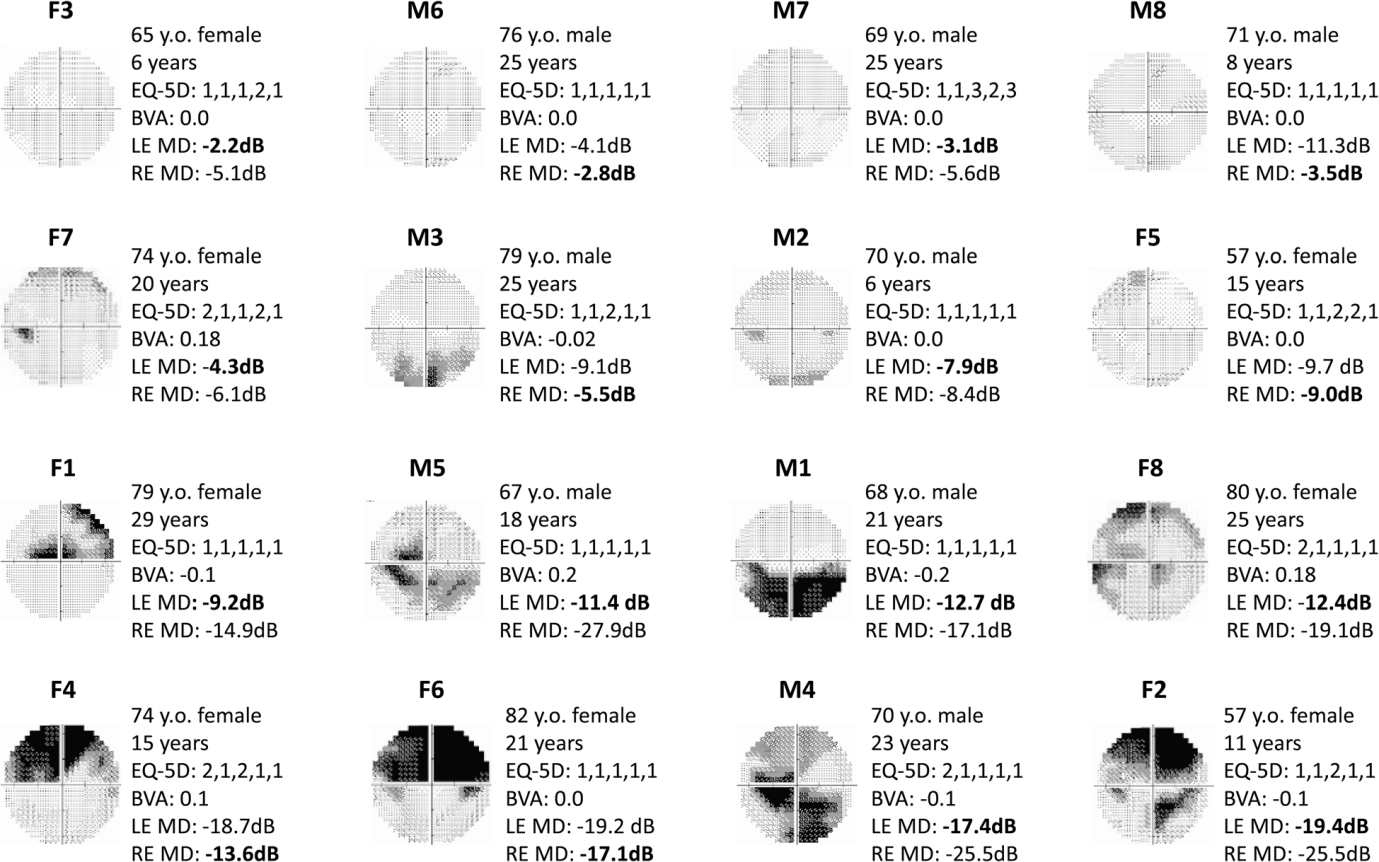
Information about each of the participants who took part in the study. The greyscale image depicts the person’s binocular integrated visual field (IVF). The information to the right denotes the participant’s age, gender, years since diagnosis, EQ-5D (3 level) health state (the scores for each of the 5 questionnaire items are shown), binocular visual acuity (logMAR), Mean Deviation (MD) for the left eye visual field (VF), and MD for the right eye VF respectively. The participants are ordered according to their best eye MD (*in bold*)

Of the 25 participants who responded to the initial advertisement and requested further information, appointments were booked for 18 to take part in the study, two of whom subsequently cancelled their appointments due to ill health. Sixteen participants (50 % female) eventually took part in the study. Patients had a median age of 71 (interquartile range [IQR]: 68 to 77) years and had been diagnosed with glaucoma for between 6 and 29 years (median: 21 years). Participants had relatively good corrected binocular visual acuity (BVA; median BVA: 0.00; IQR = −0.040 to 0.025) but a wide range of VF loss: median HFA “Mean Deviation (MD)” for the better eye (i.e. the eye with the better MD) was −9.1 dB (IQR: −12.9 to −4.1 dB). All participants scored 26 or above on the MMSE, suggesting they had normal cognitive function [24]. The participants were largely well educated (minimum level of education was secondary school, although several held higher education qualifications). All participants had previously been employed, but only two were still working at the time of the study. General health was measured according to the 3-level version of the EQ-5D questionnaire [25]. As per standard methods, health states were calculated for each participant based on their responses to the questionnaire’s five items which assess perceived difficulty with mobility, self-care, usual activities, pain and discomfort and anxiety and depression respectively. Each item has three possible responses, with a score of one indicating no problems; a score of two illustrating some problems and a score of three depicting extreme problems/unable to perform. For example, a state of “21133” would indicate “some” problems with mobility, “no” problems with self-care or usual activities, and “extreme” problems with pain/discomfort. Likewise, a health state of “11111” would indicate no perceived problems with the five health dimensions, and “33333” extreme health problems. Participants generally reported good general health, citing few perceived problems within these domains; however, one participant (M7) claimed to have “extreme” problems with performing usual activities, and to be “extremely” anxious and depressed (scoring “three” for these items). On further discussion it emerged that these issues were attributed to other health complaints, including severe allergies that were unrelated to glaucoma. More detailed information about each participant’s health state and other key variables can be found in Fig. 1.

## Results

**Fig. 2:**
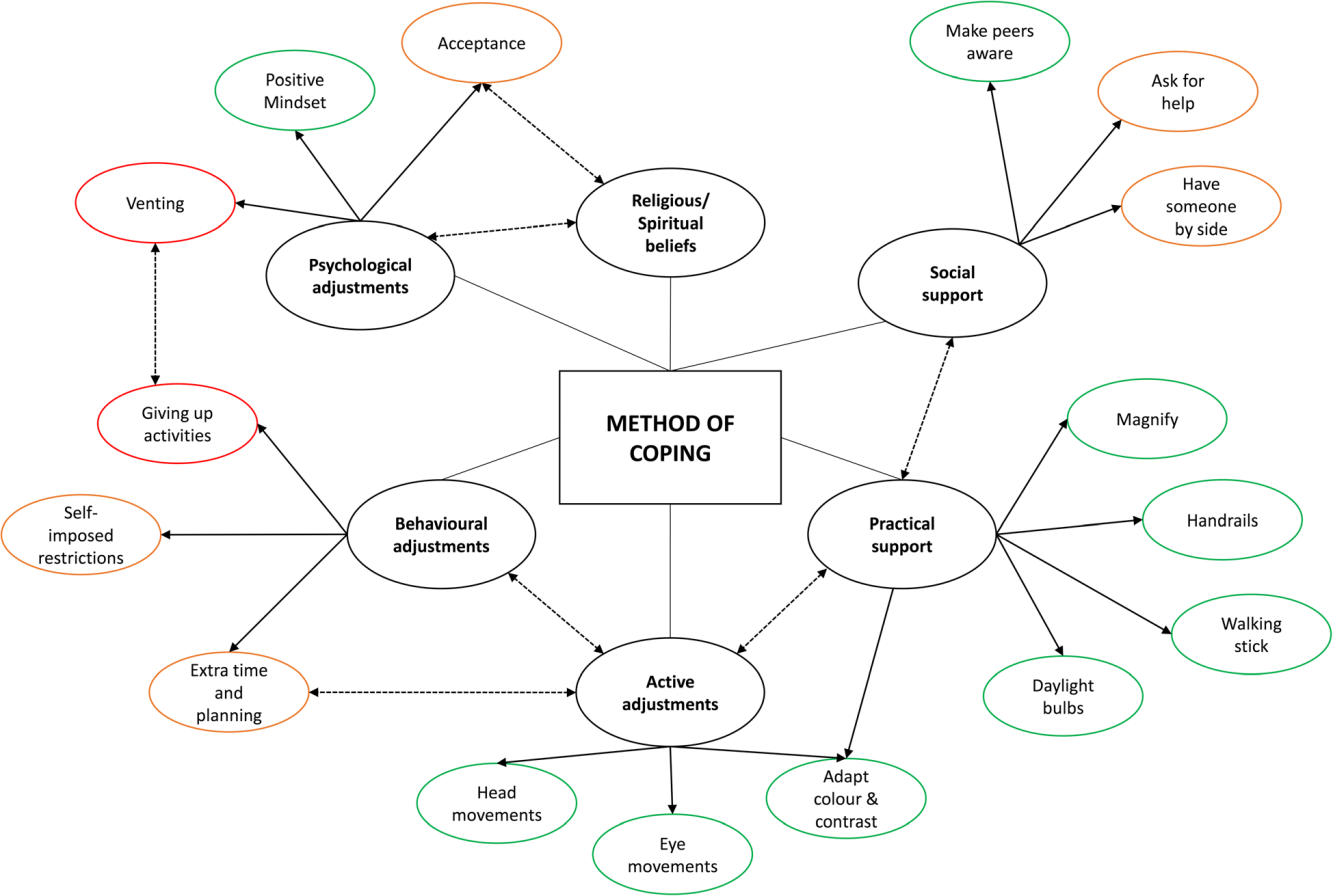
Diagram summarising the different coping techniques used by patients with glaucoma in this sample in response to problematic areas of functioning in their everyday lives. These have been coloured-coded according to whether they had a positive (*green*), neutral (*orange*) or negative (*red*) impact on perceived ability to function independently

Data was organised according to the different areas of functioning impacted by glaucoma. Within each functioning area, a number of coping behaviours and techniques emerged. Direct quotes taken from the transcripts are italicised. These quotes were examples chosen to illustrate the key themes that emerged from the interviews. All included excerpts are annotated with a code given to the corresponding participant based on their gender and the order in which they were interviewed. The key areas of functioning that were affected by glaucoma according to this group of patients are shown in Table 1. Figure 2 summarises the different coping behaviours used by the interviewed patients across the different areas of functioning.

**Table 1:** Summary of areas of functioning that were perceived to have been affected by glaucoma in this group of patients

Affected area of functioning	Examples
Activity participation	Eating and drinking, washing, shaving, reading, making art, face recognition, watching TV, using technology
Moving around environment	Walking, driving, avoiding obstacles, uneven ground, crossing roads, walking up and down stairs, manoeuvring in crowded places
Ability to uphold personal responsibilities and commitments	Social (e.g. committee membership, regular meetings); occupational (maintain job responsibilities; domestic responsibilities (e.g. housework, maintaining home, looking after family))
Importance of lighting	Brightness and glare, sunlight, manoeuvring in dim/dark conditions, adjusting from light to dark
Emotional and psychological impact	Fear of blindness; feelings of frustration, anger, disappointment; perceived control; degree of acceptance; perceived burden on friends and family

## Activity participation

When asked to describe instances in their everyday life where their vision loss became apparent, the interviewees listed tasks ranging from basic activities of daily living (washing, dressing, shaving, eating and drinking) to more complex visual activities such as reading, painting and using technology. These problems were approached in various ways: for example, some patients limited their participation in a certain activity, or avoided it altogether, due to the difficulties they had experienced, which led to feelings of frustration and resentment.

***Reading the paper or something of that nature I would just give up and say, you know, it’s not worth it. (M2)***

**

***I’m reading less because I find it’s quite hard to concentrate for long periods…(F2)***

**

***I was going to do art.. [but] there’s no point at all because …it’s not fun when you produce something that is ugh. So that’s a bit sad. (F3)***

Others were more accepting of the situation, feeling that difficulties were just something they would have to learn to live with, even if that situation was not ideal.

***The time I’m most conscious is watching the television. I find that slightly difficult… The visions aren’t as crisp as they should be… You just learn to live with it and then it doesn’t register….[but] some of the television programmes I watch, I get a bit disappointed with my vision. (M2)***

**

***Well, I paint [but] I can’t paint as well as other girls in the class… I can’t do details …[my style] is very loose. I can’t do architecture or anything like that. (F1)***

Difficulties were generally more apparent for patients with advanced VF loss in both eyes. Although some patients with unilateral vision loss were aware of their restricted VF in more contrived situations (e.g. when actively closing their eye during a binocular task), they did not notice their glaucoma in their everyday life and felt able to function normally.

***If I’m watching television I can look in that corner and I can’t see the person’s face if it’s a small face with my right eye. I’m aware of that.***

***Interviewer: So you cover up your left eye?***

***Interviewee: Yeah I just do it because I’m looking at the telly. But when I’m looking at it with both eyes there’s no problem at all. It’s only that I’m inquisitive, if you like, and I keep testing it. But if I didn’t test it I wouldn’t know and I wouldn’t know now that there was anything wrong. (M6)***

**

***I think my right eye compensates for [my poorer left eye] because sometimes if I’m reading small print I’m sure my right eye is doing most of the work. Because if I close my right eye then of course all the letters overlap, sometimes depending on whether it’s particularly bad at any given moment. (M8)***

Some patients who were experiencing difficulties when embarking upon certain everyday activities made use of instrumental support or vision aids to help them to continue to perform tasks. These were generally not sophisticated or expensive, instead involving simple practical adjustments.

***if there’s writing on a page I very often miss the top two lines. I have to get a piece of paper, [put it underneath the line] and go down if I’m reading so that I do not miss the top two [lines]. (F1)***

**

***I read a lot, both books and on [an e-reader]… Clearly one of the advantages of an e-reader is you can adjust the size of the text. I adjusted it to a larger size than was the default one. (M3)***

**

***I’ve also got a little torch so that I can shine it and see if necessary what’s – if you drop something, you can’t actually see it. (F4)***

**

***I was actually wanting to make a telephone call and… I got the magnifying glass so that I could see the number then and then I wrote it out in large figures and then I was able to get the number quickly. (F8)***

Others described active ways of adjusting aspects of their behaviour to compensate for difficulties, such as exaggerating their eye and head movements, or paying more attention to their other senses.

***… you’ve got to be careful when you’re shaving you don’t cut yourself or something with the razor. That I do find it quite difficult… I keep on having to move my head. (M7)***

**

***Interviewee: I listen to when I’m pouring water in. That’s one thing, you can tell when it’s coming to the top.***

***Interviewer: By listening?***

***Interviewee: Yes…So your other senses are kicking in. (F1)***

Certain strategies were intertwined with an understanding of problem triggers. In particular, problems seemed to be exacerbated in certain situations, such as when an item and background were of similar colour or contrast. Interviewees described different tactics for dealing with such issues, like asking for support from peers, or making conscious decisions not to place similarly coloured items together.

***So if there’s a bottle of wine and it’s heavy I’ll say, “no, you pour it”, because I’m scared of missing the glass. (F5)***

**

***Cutting brown bread on a brown cutting board, I don’t always get the thickness of the bread right. I have to look and doubly look and look at it from all angles, make sure I’m doing it okay. (M4)***

**

***The other thing with sight is the difficulty of finding things. On a patterned surface I have great difficulty finding things. If I’m putting something down I make a conscious decision o put it against a contrasting background. (F7)***

Difficulties were also apparent in more social contexts; for instance, with recognising faces.

***I’m not very good on identifying people’s faces. That’s got worse over the years, particularly on TV as well. I lose the plot very easily because I can’t figure out which character is which; I get them muddled up. Whereas my wife always knows who’s who…She has to explain that’s so and so’s wife and this, that and the other… (M4)***

**

***I know quite a few people who live locally. But I feel that if I’m a certain distance …I’m probably not going to be able to recognise them because I don’t see enough detail in their face. (M5)***

## Maintaining personal responsibilities

Although the majority of the patients interviewed in this sample were retired, there was an acknowledgement that glaucoma may have had a greater impact on their lives had they still been working.

***If I was still doing the same job that I was doing at 65 I don’t think I would be able to do it. Because I wouldn’t be able to do desk work very easily and I wouldn’t be able to use the laptop. So I think if – now the glaucoma at 70, if I had it early 60s, I would have thought at some stage I would have been not able to continue my work. (M2)***

**

Others had been motivated to give up social responsibilities, not necessarily due to the vision loss itself, but due its potential implications, such as having to go to clinical appointments at short notice.

***I’ve sort of stepped down from positions of responsibility really, being on committees and that sort of thing, because I just felt I was really unreliable and I knew I was going to have to go in for more surgery or something. That really rocked me. I became a patient. So for better or worse I drew back from doing things with any responsibility attached. (F3)***

## Moving around environment

Similar themes emerged when discussing topics relating to moving and interacting with people and places. Some patients indicated a tendency to limit the time they spent in certain situations, or avoid certain circumstances (e.g. crowded spaces) altogether, whilst others relied on social support to get from place to place. For some, an emotional burden was apparent.

***I [won’t walk] in a crowded area. (F1)***

**

***A situation where my vision does let me down a bit is if I’m in a big strange space where there’s a lot going on… where there’s a lot of busyness and a lot of detail and you’re looking out for signs or looking for something. These days I feel a bit apprehensive in a way that I would never have done in the past. I don’t really have a reason to be apprehensive, because there’s never been a problem, but somehow it just takes me a bit longer to orientate myself or find what I’m looking for.*(F2)**

**

***I really hate asking people for lifts and I’m a bit far out of town to accept lifts more than once or twice. I’m too mean to take taxis. So that’s a dilemma thing. (F3)***

The more effective strategies appeared to require an in depth understanding of the specific areas of vision that were affected.

***If I’m concentrating on one area, then something that’s just a bit to the edge of my vision disappears. It’s like I haven’t got the breadth of sharpness and vision across my visual field. It’s definitely, definitely worse on the right side than the left. Because of that I notice when I’m out walking I will sometimes just tilt my head a little bit or I need to move my head a bit more in order to be able to make sure I’ve taken in everything accurately. (F2)***

Some animated discussion took place around the topic of driving. Some patients had been required, by law, to give up their driving license, leading to feelings of frustration and disappointment relating to a loss of independence. Others recognised their ability to drive was somewhat impaired and had chosen to limit their driving to only situations where they felt comfortable, or to stop driving altogether. Some planned their day around public transport, although there was an acknowledgement that this would not be possible for everyone.

***I feel strongly about it, rightly or wrongly, I think it’s … emasculating – you know, for a man to lose his driving licence….I’d been driving for 45 years…. I’m a very bad passenger because my wife has to drive me all the time. (M1)***

**

***We’re using public transport now, mostly…It’s safer, it’s less hassle and it takes strain off my husband who has to do any driving at night or day… So we are adapting. (F4)***

**

***I don’t drive in the dark is one of the main ways I’ve adapted, in that I just don’t feel safe, I haven’t done for some time. I find the bright lights of oncoming vehicles very disturbing, so I decided probably four or five years ago that I just wasn’t going to drive at night.*(F5)**

For those still driving, eye and head movement adjustments were used as a mechanism to “take extra care” or to compensate for lack of vision when driving. For some, the nature of the adjustments were specific to a certain part of the VF, thus requiring an in depth understanding into the characteristics of their own VF loss.

***I do notice that I’ve consciously got to move my head to the right and left more. I’m saying more than I did before, because I’ve got to be extra careful. (M7)***

**

***People used to comment [when I was still driving] and said that “anybody would think you’re still taking your test, the way you move your head around” (M1)***

**

***I’m very aware, when it’s even good weather and I’m driving, I just keep my eyes moving all the time. Because I know that I’m seeing less out of the left eye. So I have to keep looking and making sure I’m judging the left hand side. (F4)***

Awareness of specific locations of VF damage was also linked to the types of strategies chosen when moving around on foot.

***[When going] up and down stairs, I actually turn my head up and down, because I’m not - I suppose I’m conscious now that I can’t see up here. I don’t know how much down here I can’t see. But I’m conscious up here and sideways a bit. (F6)***

There was some indication that certain coping behaviours may not evolve until the person had experienced at least one adverse event relating to their vision loss.

***So, I think there are minor adjustments that once you’ve done it once like hitting your head on the cupboard, I think, right, I’ve done that once, I won’t do it [anymore] or I’ll get caught on that again. (F6)***

Acceptance of particular problems was important: for instance, acknowledging the need to “take extra care” when manoeuvring steps and stairs. As such, some journeys required forward planning. Furthermore, some patients had identified a need to make use of practical support, such as handrails and walking sticks, to avoid vision-related trips and falls and maintain independence. Being accompanied and having the support of peers was also seen to alleviate some problems associated with mobility, particularly on more hazardous walking routes.

***Crossing the road is another one I think. You have to look and look again to make sure there’s nothing coming. (M4)***

**

***I’ve noticed just going up and down the subway steps on the underground, I always cling to the rail. If the steps are even, once I’ve got going I’m absolutely fine, but when you get to the bits with the bends where the steps get uneven, I’m a bit self-conscious on that, yes. (F5)***

**

***I’ve been given a folding stick so that I can hold it in front when I’m on my own - if I’ve got my husband or my son with me it’s different. I want to keep as independent as I can. (F1)***

**

***I find that if I’m leading a walk for our rambling group, the group tend to sort of rally round. It’s not always required but if we’re going over rough ground or stepping down they’ll say “Watch the branch coming up!” (M4)***

Adjusting fixation downwards towards the floor in order to avoid obstacles or to prevent tripping on uneven ground was another necessary behavioural modification. However, sometimes doing so had a negative consequence in that people would miss important information in other areas of their vision.

***These days of terrible uneven pavements and great holes, it’s quite a dangerous thing. So I have to walk with my head down to see where I’m putting my feet. (F4)***

**

***I mean - if there’s something overhanging, a tree overhanging I have walked into that because - I suppose I’m looking down. (F6)***

**

***The only time I would say I’d notice was if you’re just going on walks or you’re focused on something, I think a tree hit me in the eye or something and I’d not seen it, somewhere on the right-hand side at the top. Somehow I seem to be missing stuff up there. (F5)***

Instead of looking at the ground, others specifically adjusted their gaze in order to compensate for known issues in the VF. These strategies may have been developed over time or were suggested by a health professional based on clinical knowledge of that person’s condition.

***Well, because my left eye is my stronger eye … I would tend to turn my head just a little bit to the right so that I’m using my left eye a bit more, or look a little bit further ahead along a path. Although I obviously want to look where I’m about to put my foot next, actually I get more of a broader scope of vision if I just look a bit further ahead and use my left eye more. They’re very subtle things. (F2)***

**

***So I’ve been told to duck when I go under trees. The other things is where common-sense fits in. You’re obviously going to protect yourself. I always walk carefully. Walking today in London, I don’t want to fall. (F1)***

Letting friends and family know about specific issues was also seen as important so that assistance could be provided if necessary.

***My wife or friends always say “there's a step down” and then that helps if I know there’s one there and I can gingerly take it, particularly if it’s a dark pub. (M4)***

## Importance of lighting

Certain problems were exacerbated in particular lighting conditions. Some patients were simply accepting of the fact that lighting was an issue, whilst others relied on support, particularly in dark and busy environments. Planning ahead or allowing extra time helped compensate for troubles with poor lighting.

***I was going to say sunlight. This light’s all right but when I’m out if you get caught on the sun it actually blinds you. I can’t see anything. (F1)***

**

***Well it’s going from light to dark, like entering a cinema, I’m absolutely blinded; I can’t see anything. I have to hold onto somebody or I shall fall over any steps and I shan’t find the seat. (F7)***

**

***If I’m with other people going into the cinema, for example, I have to stop as soon as I get in…. I can’t see anything. I have to take my [time] for a few minutes so I can see enough I guess. (M5)***

Others made adjustments to lighting in their homes, relying on additional artificial lighting, such as daylight bulbs, for certain activities. However, making the effort to do so meant admitting a problem, which came more easily to some patients than others.

***In dull light and things like this I found it quite hard to see. Now our house is filled with daylight bulbs which made a huge difference. So I can actually see what I’m doing. But the light is - it’s crucial. (F4)***

**

***Interviewee: I’ve got this strong light, sort of a really good intense light for doing some sewing at the moment. So I try and remember to use it.***

**Interviewer: Do you find it helps?**

***Interviewee: Yes it does. I should use it more. But I suppose there’s also this thing, it’s easier not to do something because it’s admitting you’ve got a problem. Do you see what I’m getting at? (F3)***

## Emotional and psychological impact

There was some variation in terms of the effect of glaucoma on emotional functioning and an acknowledgement that different people deal with the condition in different ways.

***I’ve got a sister-in-law who’s just been diagnosed within the last year and she’s actually gone to pieces and she’s the last person you’d have thought would have gone to pieces…I asked her son last night … and he said he wished she’d been [as] positive [as me] because she’s negative. (F1)***

Some expressed feelings of frustration and disappointment at not being able to carry out activities in their everyday life as well as they used to. Likewise, poorer vision led to feelings of embarrassment in public situations or worry about being a nuisance or burden on friends and family.

***When I’m in church sometimes I can’t see the overhead projector or the hymns on a board if they’re not clear. They give me a sheet. It’s embarrassing because sometimes I can actually see them but they still give me the sheet (F8).***

**

***I really hate asking people for lifts and I’m a bit far out of town to accept lifts more than once or twice (F3)***

The extent to which glaucoma had an emotional impact on the patient appeared to differ depending on the length of time spent living with the condition: for example, diagnosis initially lead to feelings of fear, which were then replaced with a more reasoned perspective after a few years spent living with the condition. Some patients noted experiencing a range of emotions over time, including denial in the first instance and then anger, moving towards a more general acceptance of the situation.

***I was in big denial when they said you’ve got glaucoma. I said no I haven’t. What are these stages they say you go through? Denial, anger and all this stuff. So I was really big into denial. (F3)***

**

***….On the first day I came home and I remember going to bed and leaving the door open because I thought in the morning if I couldn’t see, the door was open. I was very fearful at the start…***

***Interviewer: So If you were to meet a person [now] who’s just been diagnosed with glaucoma, what would you tell them?***

***Don’t worry about it. Don’t worry. No problem at all. Have I got any worries about going blind or whatever, and honestly I’ve got none because the thing [hasn’t] progressed over 23 years. (M6)***

Religious and spiritual beliefs also influenced approach to life and the problems associated with disease. Some patients were accepting of their situation, perceiving glaucoma to be out of their control and something they had to live with. Others were determined to find ways to turn their situation into a positive.

***I think I’m very philosophical about these things. If you’ve got something you’ve got it. (M8)***

**

***It’s an addition that you live with, you learn with, and [think] please God hold it at bay [so] that it doesn’t get dramatically worse (F7)***

***I think mainly I just try not to let it stop me doing anything. (F5)***

**

***The answer is, you do what you can now and go for it. I guess that’s the bottom line. Every day’s special, that’s the only way to look at it. (F4)***

## Additional themes

Some additional themes also emerged during the analysis. For example, the importance of considering these matters according to the wider context of a person’s history and home life was emphasised. Some patients had also experienced other long-term medical conditions (such as hearing loss, cancer and allergies) and they discussed the relevance of glaucoma to their lives in comparison to these other health issues. It was also seen as important to consider the person’s close social network and the health and situation of close family members such as one’s spouse.

***I think most people are very helpful on the eyesight side. They’re not so helpful on the hearing side. They’re not so understanding. I think probably this comes from - in England, we have a general awareness of blindness, white sticks. So when you say to somebody, oh, have you got sight problems? People are very aware of it. But deafness does not get the same empathy at all. (F4)***

**

***With all my allergies and intolerance…. with all my problems - even so, I’m more worried about glaucoma than anything because you can’t cure it, you can only hope it doesn’t get worse. (M7)***

Patients also drew attention to their experience of patient information and education, particularly the explanations about glaucoma given at diagnosis. When first told they had glaucoma, the information received by patients tended to focus on the nature of the disease itself, rather than how it would impact on their life. Some had received little reassurance, instead being told that they were destined to go blind if they did not “do what they were told”, which caused initial feelings of alarm and anguish. Limited information was also provided about how glaucoma might manifest itself in everyday life, even causing speculation that this information was being held back on purpose.

***They said its retinal death of the optic nerve and this is really serious, it’s really horrible, but you’ll be fine if you just take your drops…But you can’t find anywhere how it will manifest itself. Maybe…..is this going to be so ghastly nobody dare say what it is. This feels like there’s a conspiracy of silence, but maybe that’s just medics for you, I don’t know. (F3)***

***If people realise how much it would affect them, job-wise let alone their social life, I think that’s the only way you bring it to people’s attention for them to take notice. How it’s going to affect them personally. (M1)***

Clinicians who had taken the time to talk about their patient’s condition in a more relevant and understandable way received respect. Having their condition explained using meaningful metaphors and examples relevant to their individual disease characteristics was helpful.

***The gentleman I saw first,…he likened it to going down a road - he said you can’t go left or you can’t go right, if you do as I tell you, you won’t go blind….***

***[as a result of doing the VF test] I know that the top half has gone on both eyes. (F1)***

Some patients found it helpful to discuss and share experiences with others in a similar situation, but this was not always possible due to limited opportunities to meet others with glaucoma or a lack of support groups in area. Speaking to friends and family about their particular difficulties and concerns was an alternative approach.

***Going right back to the start [when I was first diagnosed], I think it would have been a great help if I could have talked to someone who’d had the same situation. (M6)***

**

***I suppose I don’t know anyone, there’s been no one in the family [with glaucoma] and there’s no support groups [where I live] or anything, there’s no practice nurses you can talk to.***

***Interviewer: Would you have found that helpful?***

***Interviewee: I think it might have been helpful to begin with, especially when I was in my strong denial phase. [F3]***

**

***Do tell all your family and that you - what you find difficult? So that you do get some help from people saying, there’s a flight of steps here, there are two steps there… (F4)***

It is worth noting that patients in this sample enjoyed doing their own research, and emphasised the need to be proactive and seek information and answers to questions.

***I would like to emphasise that to people. The fact that you have got to be in the controlling seat… nobody’s going to do the job for you, you’ve got to do it. (F4)***

## Discussion

Recent research activity suggests that glaucoma can have a significant impact on the everyday lives of patients, yet much less attention has been given to the ways in which a patient might cope or adapt to their vision loss. This study was the first, to our knowledge, to directly explore these matters in tandem. Interviews were conducted with a sample of glaucomatous patients with a wide range of VF loss. Although some patients claimed they were largely unaffected by their glaucoma, several areas of functioning were deemed problematic, including, for example, reading, and difficulty with face recognition. Patients also reported issues relating to mobility (walking and driving), navigating around obstacles (including steps and uneven ground) and their general interaction with people and places. The importance of lighting was a strong theme. Moreover, emotional, as well as physical implications of vision loss were acknowledged. These areas coincide well with those identified during development of other measures of glaucoma-related functioning [26–29].

The extent to which patients’ functioning was affected and how they approached any perceived problems varied greatly. For instance, some tackled perceived issues by making use of more instrumental approaches (e.g. adjusting lighting, using handrails, and magnifying glasses) or by taking extra time to plan for events. Actively modifying aspects of behaviour, such as adjusting eye and head movements to compensate for areas for vision loss was articulated. This finding implies that previous research findings showing that patients use different eye movement strategies during visual tasks may have a conscious component [9, 12, 30]. Some patients relied on the support of friends and family, but this sometimes led to anxiety about loss of independence or becoming a burden. Psychological and emotional responses to vision loss also varied between patients: some people simply accepted their condition, whilst others made an additional effort to reassess their situation in more positive light. Others responded more negatively, disengaging both mentally and physically from their environment which led to the expression of emotions such as anger, resentment or disappointment. These behaviours emerged across most of the functioning areas, suggesting they were not necessarily task-specific and were instead a more personal response to glaucoma as a whole.

Type of coping was somewhat related to specific characteristics of their vision loss. The patients in this study had been diagnosed with glaucoma for varying lengths of times (between 6 and 29 years) and there was an indication that the time spent living with glaucoma may have influenced how patients perceived their situation. Some patients noted feelings of fear and despair when diagnosed, but changed their attitude after actually living with the condition. For example, patient M6 reported no functional symptoms because he had negligible VF loss. Yet M6 eloquently described how he experienced an intense fear of blindness on diagnosis, specifying that it took over 25 years of living with glaucoma (that did not progress) to be reassured about his vision. F3 on the other hand - diagnosed 6 years ago - described feelings of denial and anger, and had since given up a number of activities because of her perceived vision loss. Interestingly the clinical measures of vision in these two patients were very similar (see Fig. 1), suggesting they had employed different adjustment approaches, or indeed that they had progressed to different stages of a wider adjustment process experienced by all patients, such as those described in the well-known Kubler-Ross model for grief [31, 32]). Regardless, this finding appears to illustrate the impact of time since diagnosis and the need for an understanding about progression of disease. It also highlights the importance of information at diagnosis.

Likewise, specific knowledge of the location of VF loss being chiefly affected also appeared to be beneficial: one patient (F4), diagnosed 15 years ago, appeared aware of the parts of her vision that were poorest, and had subsequently developed a number of active coping behaviours, such as consciously making an effort to move her eyes towards affected parts of the VF. She had also adapted the lighting in her home, asked friends and family for help and made use of public transport. It is worth noting that the adoption of these strategies may have arisen from an innate desire to, by her own admission, *“be in the controlling seat*” and an inclination to do her own research and ask questions. In reality, however, many patients do not receive feedback about their vision during their clinical appointment unless they ask for it [33]. The manner in which key health information is communicated may influence a person’s overall understanding of their condition and how they approach aspects of their management. For example, the way in which clinicians communicate with the patient can influence future adherence to treatment [34]. Many of the patients in the current study expressed some concerns about the relevance of information they had received during their clinical appointments, commenting that it would have been useful to have been told more about how glaucoma might manifest itself in their everyday life. This idea is corroborated by the experiences of patient F1, who had been told by her clinician that her superior (upper) vision was affected, and thus was confident she should avoid potential hazards within this location. The study findings also reinforce the idea that some coping strategies are more successful than others. For example, several patients described a tendency to maintain a steady fixation on a particular region, such as focusing on the ground when walking, meaning that they could miss other important information in the visual field. These findings correspond with other eye movement research that suggests that some patients with glaucoma display more restrictive scanning strategies, which could have negative knock-on effects, such as causing them to miss potential hazards when driving [10, 35]. Some other behaviours displayed by patients in this study, such as denying the extent of their condition, or limiting their activity participation, could also be perceived as being maladaptive (Fig. 2). Quantifying the relative effectiveness of specific strategies and behaviours may be necessary for future studies and educational approaches.

The idea of being able to share experiences with other patients, particularly at the earlier stages of treatment, was reacted to favourably in the current study, although this was not seen as achievable for some due to a lack of support groups or facilities in their area. Studies have highlighted some positive effects from social support and buddy systems for improving care delivery for other conditions, suggesting this may be an approach worthy of investigation for glaucoma care [36, 37]. Effective translation of information from more “expert” patients, who have lived with glaucoma for a period of time and appear to have a good understanding of their condition, could be an additional approach for patient management and this study serves as stimulation for future work in this area. Future studies may also wish to consider the link between onset of disease and adaptive behaviours, and consider the value of providing education at diagnosis to prepare participants for future challenges.

The potential influence of memory-related biases on the results should be acknowledged. We screened the participants using the MMSE to ensure that they did not show signs of dementia; however, this was not an in depth cognitive assessment and the retrospective nature of the study meant that we cannot be sure that participants’ accounts were entirely accurate or free of other subjective biases. The exact influence of visual characteristics remains unclear: for example, the range of visual field defects included in this sample makes it difficult to decipher the exact nature of the role played by visual field loss in comparison to personality-related factors or general responses to the normal ageing process. Other potentially important visual factors, such as near vision or colour vision, were also not measured. Likewise, the findings of this study might not generalise to a wider population. This study used purposeful sampling, in that participants were members of a patient-based charity for glaucoma who volunteered to talk about their experiences. Most of the participants were able and willing to travel to London to take part in the study. Furthermore, most of the participants were proactive and inquisitive by nature- they had joined a charity, read newsletters, asked questions and sought information about their condition and were likely better informed about glaucoma than the general patient population. As such, they may be more aware of any conscious strategies they have developed than other patients. Whilst this is useful from an educational purpose, future research should also consider the relationship between functioning and coping in a much larger and more representative population. Likewise, the importance of considering the wider clinical and personal context of the person when interpreting results should be acknowledged: for example, some of these patients were also affected by (non-ocular) co-morbidities, often age-related, which influenced their perspectives. Future work may also wish to focus specifically on how adverse events or side effects caused by glaucoma treatment may influence patient perceptions of their condition. Cultural influences are also likely to impact subjective wellbeing and how people adapt to a condition [38]. Some of the participants in this study appeared to have strong religious and spiritual beliefs- factors that are likely to strongly influence the manner in which a person approaches their condition and life in general.

This is not the first study to interview patients with glaucoma about their experiences: prior research studies have also endorsed the use of qualitative methods for improving understanding into the impact of glaucoma from the patient’s perspective. For example, previous research has asked patients to describe what vision loss “looks like”, challenging simple depictions of the visual symptoms of the disease [15]. Studies have used focus groups to explore patients’ viewpoints about reasons for late diagnosis [1], aspects of their follow-up care [33], and perceived barriers to treatment adherence [39]. One similar study, conducted over a decade ago, conducted individual and group interviews with patients with glaucoma about what it is like to live with glaucoma [14]. Patients reported that they experienced few negative effects of glaucoma initially, but had to learn to live with the condition as it worsened over time. The authors called for improvements in health education to better raise awareness of symptoms and events that are relevant to the experiences of patients. More than ten years on, and in an era with a much more patient-centric focus for healthcare [40], it is surprising that there has been little attempt to build on this work. Recent research has highlighted the potential of a more individualised treatment approach for improving aspects of glaucoma care [41, 42]. Providing tailored education and sharing ideas about incorporating management into everyday living has been found to be beneficial for other conditions [43, 44]. There may therefore be scope to build on this work by devising interventions that teach patients coping techniques and assess impact on perceived self-efficacy and quality of life.

## Conclusions

Glaucoma can impact on a person’s life across multiple domains. This study confirms the highly variable between-person responses to living with glaucoma but also serves to highlight strategies adopted by patients in an attempt to compensate for their vision loss. Active strategies, such as making use of practical support or consciously making head and eye movements towards areas of vision loss, were noteworthy in this sample of patients. A holistic approach to vision assessment in glaucoma, highlighting coping and adaptive methods, in addition to clinical tests, may ultimately provide a better insight into the impact of disease and help inform future educational and management strategies.

## Abbreviations

CAQDAS: Computer Assisted Qualitative Data Analysis Software
COREQ: Consolidated criteria for reporting qualitative research
CS: Contrast sensitivity
EQ-5D: EuroQol 5-Dimensions
HFA: Humphrey Field Analyser
IVF: Integrated visual field
MD: Mean Deviation
MMSE: Mini Mental Status Examination
POAG: Primary Open Angle Glaucoma
VA: Visual acuity
VF: Visual field
